# Cysteine dioxygenase knockout and taurine deficiency impair mouse uterine adenogenesis by inhibiting epithelial cell proliferation and enhancing apoptosis

**DOI:** 10.1371/journal.pone.0329503

**Published:** 2025-08-18

**Authors:** Hui Liu, Yuneng Gong, Xiaoyan Qu, Sheng Cui, Di Zhang

**Affiliations:** 1 College of Veterinary Medicine, Yangzhou University, Yangzhou, Jiangsu, People’s Republic of China; 2 Institute of Reproduction and Metabolism, Yangzhou University, Jiangsu, People’s Republic of China; 3 Jiangsu Co‑Innovation Center for Prevention and Control of Important Animal Infectious Diseases and Zoonoses, Yangzhou University, Yangzhou, People’s Republic of China; Korea Institute of Radiological and Medical Sciences, KOREA, REPUBLIC OF

## Abstract

Uterine glands and their secretions are essential for conceptus survival and development, with abnormalities in uterine gland morphogenesis (adenogenesis) are closely related to high rates of peri-implantation embryonic loss in humans and livestock. While uterine adenogenesis occurs postnatally in most mammals, the key regulatory factors and mechanisms governing this developmental event remains largely unexplored. Our recent study reveals that cysteine dioxygenase (CDO) is highly expressed in the uterus of adult mice, which is also rich in taurine. Notably, *Cdo* knockout (KO) and the resulting taurine deficiency lead to the defects in embryo implantation and subfertility. However, the regulatory roles of CDO and taurine in uterine development and adenogenesis remain unclear. In the current study, we assayed CDO expression and taurine content in the developmental uteri of mice from postnatal day (PND) 3 to PND 28, and investigated the regulatory roles of CDO and taurine in uterine adenogenesis using *Cdo* KO mice. Our results showed that uterine CDO protein expression gradually increased from PND 3 to prepuberty, closely correlating with uterine taurine levels. *Cdo* KO and taurine deficiency impaired the formation and development of uterine gland by inhibiting uterine epithelial cell proliferation and enhancing cell apoptosis. Remarkably, taurine supplementation partially rescued these defects in uterine adenogenesis. These findings, for the first time, demonstrate that uterine tissue acquires the ability to synthesis taurine postnatally, CDO and taurine act as novel factors regulating uterine gland development. Uncovering the mechanisms of uterine adenogenesis could significantly improve pregnancy outcomes in humans and other mammals.

## Introduction

The uterus is composed of an outer muscular layer, the myometrium, and an inner mucosal layer, the endometrium. The endometrium provides a protective environment for the embryo implantation and development. The myometrium undergoes contractions both in the non-pregnant and pregnant states, playing a key role in guiding the embryo prior to implantation and expelling the baby during birth [1,2]. The endometrium is made up of a layer of luminal epithelium (LE), supported by stromal cells, and contains coiled endometrial glands, also known as glandular epithelium (GE). These glands synthesize and secrete proteins as well as related substances that nourish the developing embryos [3–5]. Disruptions in uterine development or the formation of GE can lead to subfertility or permanent infertility in adults [6,7]. Consequently, it is crucial to study the regulatory mechanisms underlying uterine development and adenogenesis.

The mammalian uterus arises from the Müllerian ducts during fetal development [8,9], and consists of simple columnar epithelium surrounded by undifferentiated mesenchymal cells at birth [10]. The uterus then undergoes a series of developmental events to acquire full reproductive capacity, including the differentiation of the stroma and myometrium, and the development of uterine glands (adenogenesis). Adenogenesis occurs in mice from postnatal day (PND) 3 to PND 21 [11]. By PND 5, LE cells proliferate rapidly and invaginate into the stroma, indicating the initiation of GE development. Between PND 7 and PND 9, developing glands appear as teardrop-shaped epithelial buds that progressively elongate, coil, and adopt a sinuous morphology. By PND 15, the mouse uterus acquires its fundamental structure and functional architecture, including well-organized LE, GE, and the supporting stroma as well as myometrium [11,12].

Uterine development and adenogenesis are regulated by multiple factors, including WNT signaling and WNT-related genes [13,14], Axin2 [15] PR-Set7 [12], Lgr4 and Dlx5/6 [16] and Foxa2 [17]. Foxa2, in particular, is specially expressed in uterine GE and has been identified as the marker molecule of uterine glands [17]. In addition, various factors such as lactocrine signaling [18], prolactin [19], growth hormone [4], fibroblast growth factors [20], and insulin-like growth factors (IGF1 and IGF2) [21,22] were reported contributing to the regulation of uterine development. Steroid hormones also play roles in this process, though it has been shown that prepuberty development of mouse uterus is independent of steroid hormones [23]. Thus, uterine adenogenesis is governed by a highly coordinated network of local and systemic signaling molecules and hormones. However, it is largely unexplored so far about the mechanisms regulating the dynamic epithelial growth, cell proliferation, differentiation, and apoptosis during adenogenesis.

Taurine, a highly abundant non-essential amino acids, plays essential physiological roles, including bile salt synthesis, hepatoprotection, energy metabolism, antioxidative activity, osmoregulation, and anti-inflammatory as well as anti-apoptotic effects [24,25]. The maintenance of taurine level in the body mainly relies on active dietary uptake and endogenous synthesis, which is catalyzed by enzymes including cysteine dioxygenase (CDO) [26]. CDO is expressed across multiple tissues, including the liver, adipose tissue, pancreas, kidneys, lungs and reproductive system [27]. Knockout (KO) of the *Cdo* gene results in increased postnatal mortality, impaired postnatal growth and compromised male fertility [28]. In addition, our recent study shows that CDO is highly expressed in the mouse uterus [29], and both uterine tissue and uterine luminal fluid (ULF) are rich in taurine [30]. Furthermore, CDO expression and taurine concentrations rise during embryo implantation, and taurine plays a critical role in regulating this process [29]. Despite these findings, the roles of CDO and taurine in regulating uterine development and adenogenesis remain largely unexplored. In the present study, we assayed the CDO expression and taurine content in the developing mouse uteri from PND 3 to PND 28. The *Cdo* KO mouse model was then used to assess the regulatory functions of CDO and uncover previously unrecognized roles of CDO and taurine in the regulation of uterine adenogenesis and its related mechanisms.

## Materials and methods

### Animals and treatments

*Cdo* KO mice for this study were established in our laboratory [29]. All of the mice for this study were raised in controlled temperature (25 ± 1°C) and humidity (60%–70%) with a 12 h light, 12 h dark cycle. Mice were sacrificed by cervical dislocation (adult) or decapitation (PND 3–28) after deeply anesthetized with intraperitoneal injection of Zoletil 50 (75 mg/kg, Virbac, France). The animal experiments were approved by the Chinese Association for Laboratory Animal Sciences. Adult female mice were mated with matured males to induce pregnancy.

### Real-time quantitative PCR (RT-qPCR) and common PCR

Total RNA of the uterus tissues was isolated using the TRIzol reagent (Takara, Dalian, China), purified by DNase I and quantified by spectrophotometry. 1 μg purified total RNA was used as a template for cDNA synthesis using HiScript Reverse Transcriptase (Vazyme, Nanjing, China) according to the manufacturer’s instructions. RT-qPCR was performed using SYBR Green master mix (Vazyme, Nanjing, China) in the StepOnePlus Real-Time PCR System (Applied Biosystems, Foster City, CA, USA) and reactions were done in triplicate. RT-qPCR conditions were as follows: 95°C for 2 min, followed by 40 cycles of 95°C for 15 s and 60°C for 1 min. Relative gene expressions were normalized to endogenous control Gapdh. *Cdo* forward primer-TGGAAGCCTACGAGAGCAATCC, reverse primer-AGCTTCAGAAAGCAGTGGGAGT; *foxa2* forward primer-TCCGACTGGAGCAGCTACTACG, reverse primer-CAGCGCCCACATAGGATGACAT and *gapdh* forward primer-TCCTGCACCACCAACTGCTTAG, reverse primer-ATGACCTTGCCCACAGCCTTG were used. All Primers were designed using NCBI.

The genotype identification of the *Cdo* KO mice was performed by common PCR using primers as following: CDO325 F-GGACCAACCACTGAGTTCATCT, CDO325 R-AATGAATGAGTCCAACCCTGCT; CDO245 F-GGACCTCATCCGCATCTTACAT, CDO245 R-GCGACAGAGAGCTGAAAATCTG [29]. Amplifications were carried out on PCR instrument (Bio-Rad, Hercules, CA, USA) using the following protocol: 94°C for 5 min (one time); 94°C for 50s, 65°C for30s, 72°C for 30s (35 times); 72°C for 10 min; and holding at 4°C.

### Western blotting (WB)

The uteri tissues were lysed with RIPA buffer (Beyotime, Shanghai, China) containing 1 mmol/L phenylmethanesulfonyl fluoride (PMSF, Sangon Biotech, Shanghai, China). The protein concentration of each group was determined by using the BCA assay reagent (CoWin Bio- sciences, Jiangsu, China) according to the manufacturer’s recommendations. Equal amounts of 70 μg proteins were electrophoresed on 15% sodium dodecyl sulfate–poly- acrylamide gel (SDS-PAGE), and the bands were transferred to 0.45 μm polyvinylidene difluoride (PVDF) membrane (Millipore, MA, USA). The membrane was blocked with 5% (w/v) nonfat dry milk in 0.05 mol/L pH 7.4 Tris buffered saline (TBS) for 1 h and incubated with rabbit anti-CDO antibody (ab53436, abcam, Cambridge, UK; 1:2000), mouse anti- proliferating cell nuclear antigen (PCNA) antibody (60097–1-Ig, Proteintech Group, Inc., IL, USA; 1:2000), rabbit anti-BCL2-Associated X Protein (BAX) antibody (T40051, Abmart Shanghai Co.,Ltd., China, 1:2000), rabbit anti-BCL2-Associated X Protein (BAX) antibody (ab182858, abcam, Cambridge, UK; 1:2000), and internal control rabbit anti-Tubulin antibody (K006154P, Beijing Solarbio Science & Technology Co.,Ltd., China, 1:2000) overnight at 4°C. The PVDF membrane was then washed 3 times for 30 min in TBST (0.1% Tween-20 in TBS) and incubated for 2 h with horseradish peroxidase-conjugated goat anti- rabbit IgG or horseradish peroxidase-conjugated goat anti-mouse IgG (Zhongshan, Beijing, China). After washing for 30 min with 3 changes of TBST, the membrane was treated with the ECL kit (Vazyme, Nanjing, China) and visualized by Tannon gel imager (Tanon, Shanghai, China). The relative intensity of each blot was assessed and analyzed with the Image J (National Institutes of Health, USA) Software package.

### Immunofluorescence (IF) staining

Tissues were fixed in 4% paraformaldehyde, dehydrated via graded ethanol solutions, and then embedded in paraffin to obtain 5 μm thick sections. For immunofluorescence (IF), antigen retrieved sections were incubated with 10% normal donkey serum to block non- specific binding sites at room temperature for 30 min. The sections were then incubated with CDO (1:200, ab53436, abcam, Cambridge, UK), mouse anti-α-SMA antibody (1:200, SC53142; Santa Cruz Biotechnology, USA), rabbit anti-CK antibody (1:200, ab53280; Abcam, Cambridge, UK), rabbit anti-Foxa2 antibody (1:200, #818; Cell Signaling Technology, USA) and rabbit anti-Ki67 antibody (1:200, D385; Cell Signaling Technology) overnight at 4°C. After washing, the sections were incubated with 555- or 488-conjugated donkey anti-rabbit/mouse antibody (1:200, Jackson ImmunoResearch Laboratories)

TUNEL assay was conducted according to the manufacturer’s instructions (C1088, Beyotime, China) to identify apoptotic cells. Signals were collected under a microscope (Olympus, Japan).

### Counting of uterine epithelial cells and glands

For the count of uterine epithelial cells, the paraffin-embedded sections of uterine samples at distinct developmental time points were stained using the antibody against cytokeratin (CK) to mark the epithelial cells and the nuclei was stained with DAPI. CK and DAPI double-positive cells per cross-section were counted under microscope. In addition, FoxA2 IF staining was performed and FoxA2 marked uterine glands on each section were counted. Four individual mice were used for quantification at each developmental time point and five discontinuous cross-sections of each mouse were counted.

### Measurement of taurine

The taurine contents were measured by High Performance Liquid Chromatography–UV (HPLC). Firstly, samples were weighed, homogenized and deproteinized using 0.2 mol/L sulfo-salicylic acid. After being centrifuged at 14,000 × g for 20 min, the supernatants were added into a dual-bed column containing cation exchange resins to remove other amino acids and metabolic precursors of taurine. Secondly, samples were added with 100 μmol/L glutamine as an internal standard. All samples were then filtrated through a 0.22-μm PVDF membrane and saved in −80°C refrigerator until use. The samples and the standard samples of taurine which were 100, 50, 25, 10, 5, 2 and 1 μmol/L were derivated with OPA (Sigma- Aldrich, St. Louis, MO, USA) solution (20 mg OPA, 2 mL methanol, 80 μL 2-hydroxy-1-etanethiol, 18 mL 0.1 mol/L borate buffer (pH 9.6)) for 3 min. Then 20 μL sample was automatically injected into a six-port valve to analysis with Waters Symmetry C18 Column (4.6 μm, 150 mm × 5 mm) (Waters, Milford, MA, USA) on a Shimadzu HPLC system (Shimadzu, Kyoto, Japan). The HPLC conditions were: flow A: 100% methanol, flow B: sodium phosphate buffer pH 4.7 containing 50% methanol. Flow rate was 1.2 mL/min, and the detection wavelength was 340 nm. The duration times were 2.3 min and 4.95 min for the internal standard and taurine.

### Statistical analysis

Statistical analysis was performed using GraphPad Prism 8.0. Data from at least three independent samples were expressed as mean ± SEM. Two group comparison studies were performed using Student’s t-test. One-way analysis of variance (ANOVA) was used for data comprising three or more groups.

## Results

### 1. CDO expression and its relation to taurine levels in the developmental mouse uterus

**Fig 1:**
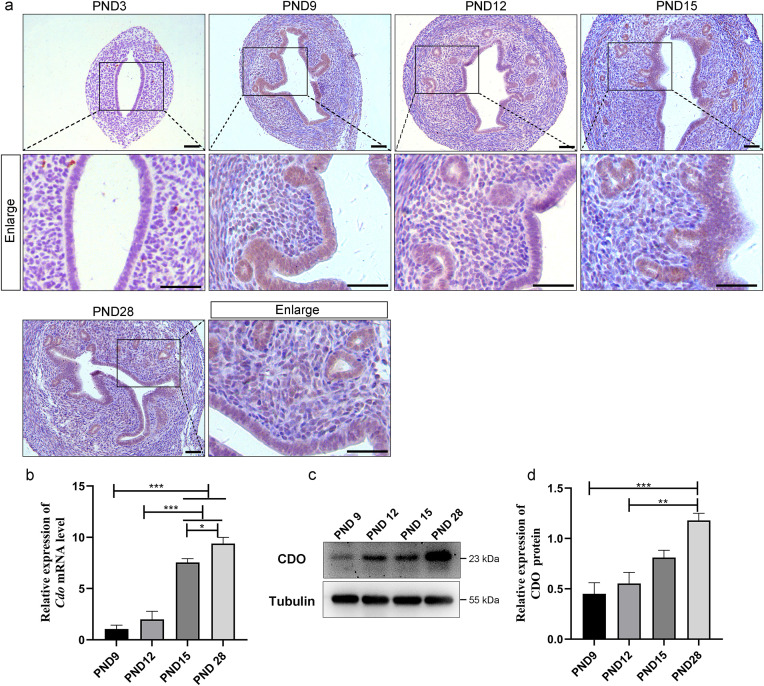
CDO expression in the developmental uteri of postnatal mice. (a) CDO immunohistochemical staining of the uteri of the mice at PND 3, PND9, PND12, PND 15 and PND 28. (b) RT-qPCR analysis of relative *Cdo mRNA* levels in the uteri of the postnatal mice at PND9, PND12, PND 15 and PND 28 (n ≥ 3). (c-d) Western Blot detection and analysis of CDO protein expression in the uteri of the postnatal mice at PND9, PND12, PND 15 and PND 28, with Tubulin as an internal reference (n ≥ 3). PND: Postnatal day. Brown signal is CDO positive signal; blue signal is hematoxylin cell nuclear staining. Scale bar represents 50 μm. ****P < 0.0001, ***P < 0.001, **P < 0.01.

We first examined the temporal expression patterns of CDO in the uteri of mice at PND 3, PND 9, PND 12, PND 15 and PND 28 by immunohistochemistry (IHC). The results showed that CDO staining was undetectable at PND 3. However, at subsequent developmental stages, CDO was highly expressed in the epithelial cells, particularly in the epithelium, including LE and GE, with weaker staining observed in the stroma and myometrium (Fig 1a). Because CDO IHC staining was negative at PND3 and uterine adenogenesis occurs after PND3, the CDO *mRNA* and protein expressions in mouse uteri were assayed by RT-qPCR and Western Blot at PND 9, PND 12, PND 15 and PND 28. The results showed that both *Cdo mRNA* and protein levels increased from PND 9 to PND 15, and the CDO protein level reached the highest expression at PND 28. (Fig 1b–d). These results suggest that the uterus acquires the ability to synthesis taurine relying on CDO after PND 3.

### 2. Characterization of the *Cdo* KO mouse model

**Fig 2:**
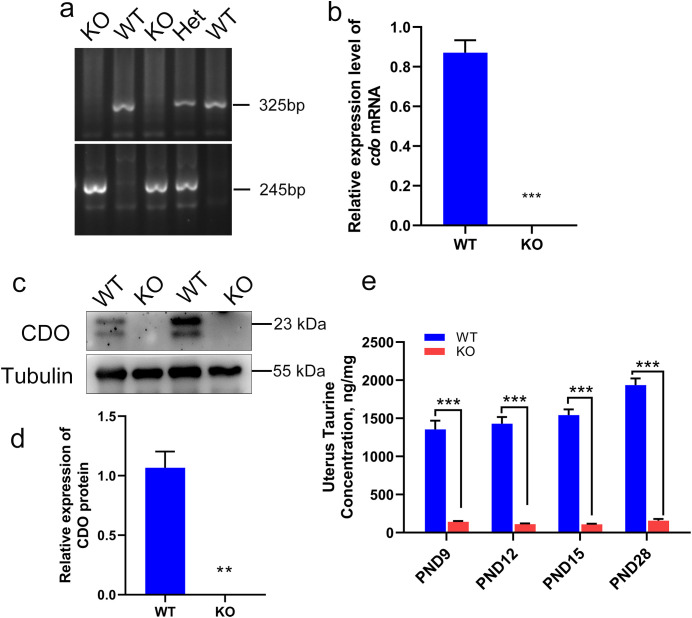
Detections of mouse genotypes and *Cdo* KO efficiency. (a) Agarose gel electrophoresis detection of mouse genotypes. (b) *Cdo* mRNA expression in the uterus of the KO and WT mice. (c-d) Western Blot detection and analysis of CDO protein expression in the uteri of the KO and WT mice, with Tubulin as an internal reference (n ≥ 3). (e) Taurine content in uterine tissue of the KO and WT mice at PND 9, PND 12, PND 15 and PND 28, assayed by HPLC. WT: wild type. KO: *Cdo* KO. Het: heterozygous. PND: Postnatal day.

In order to identify the functions of CDO and taurine in uterine development, we used the *Cdo* KO mice [29]. The genotypes of the mice were confirmed by PCR, with the 245-base pair (bp) strand representing detection of the mutant allele and the 325 bp strand identifying the wild-type (WT) allele (Fig 2a). Knockout of *Cdo* was confirmed by RT-qPCR and Western blot analysis, which showed a complete absence of *Cdo mRNA* and protein expressions in the uterine tissue of *Cdo* KO mice (Fig 2b–d). Furthermore, taurine concentrations in the developing uteri of *Cdo* KO and WT mice were measured at PND9, PND12 and PND15 using HPLC. The results showed that taurine levels in *Cdo* KO mice were dramatically reduced by 89%, 92% and 93% at these time points, respectively, compared to WT controls (Fig 2e). These results demonstrate that CDO is successfully deleted from mouse genome and lack of CDO leads to taurine deficiency in the mouse uterus.

### 3. Effects of *Cdo* KO on the mouse uterine development

**Fig 3:**
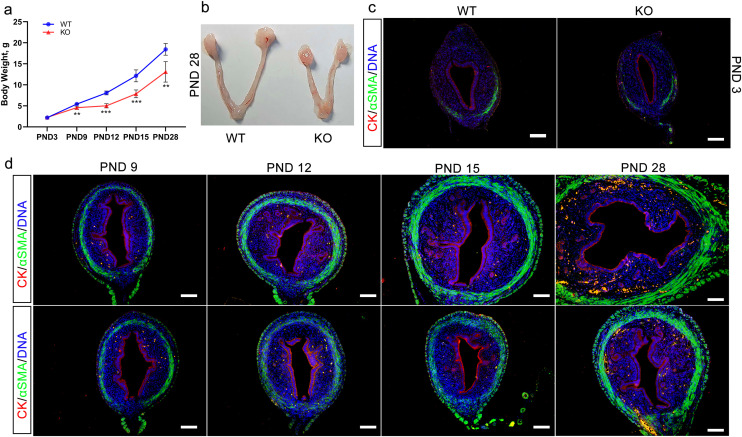
Effects of *Cdo* KO on the uterine development of mouse. (a) Body weights of *Cdo* KO and WT mice from PND 3 to PND 28 (n ≥ 4). (b) Uterine morphology of *Cdo* KO and WT mice at PND 28. (c-d) Dual IF staining of CK (red) and α-SMA (Green) on the uterine sections of *Cdo* KO and WT mice at PND 3, PND 9, PND 12, PND 15 and PND 28. WT: wild type. KO: *Cdo* KO. PND: Postnatal day. Scale bar represents 50 μm.

We first monitored body weight from PND 3 to PND 28 in both *Cdo* KO and WT mice. From PND 9 onwards, *Cdo* KO mice exhibited significantly reduced weight gain compared to WT controls (Fig 3a). By PND 28, it was observed that the uteri of the *Cdo* KO mice was significantly smaller than those of the WT mice, suggesting a developmental deficiency (Fig 3b). Next, dual immunofluorescence (IF) staining for cytokeratin (CK), an epithelial marker, and α-smooth muscle actin (α-SMA) (the marker of myometrium) was performed to examine the histological differences between *Cdo* KO and WT uteri at PND 3, PND 9, PND 15 and PND 28. At PND 3, the uteri of both *Cdo* KO and WT mice displayed similar basic structures, including simple epithelium, mesenchyme and myometrium. At PND 9, the basic structure of the uterus, including myometrium, stroma, LE and the invaginating epithelial tubes, were clearly observed, with no significant differences in structural integrity at this stage in the *Cdo* KO uterus. However, from PND 12 to PND 28, while the myometrium, stroma, and LE became clearly distinguishable, the *Cdo* KO uteri displayed obvious developmental impairments. The cross-sectional area of the uterus, and the thickness of the myometrium, stroma and uterine cavity, were significantly smaller in *Cdo* KO mice compared to WT mice (Fig 3c and d). Notably, epithelial invaginations and branching, which are essential for uterine gland formation, were markedly reduced in *Cdo* KO mice compared to the WT mice (Fig 3c and d). These findings infer that *Cdo* KO inhibits uterine development and adenogenesis.

### 4. *Cdo* KO impairs uterine gland development

**Fig 4:**
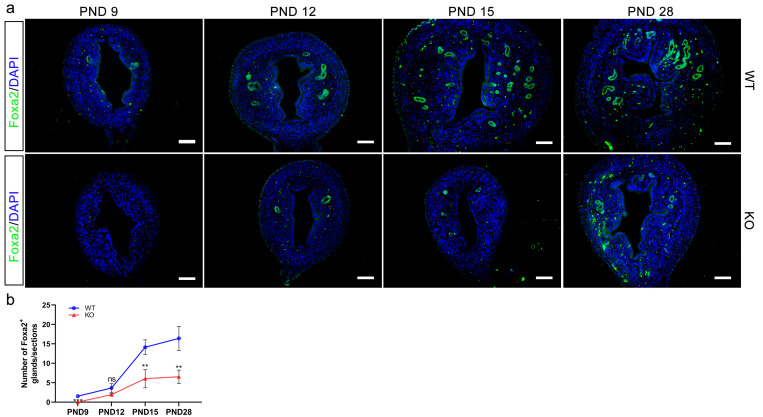
*Cdo* KO impairs uterine gland development. (a) IF staining of Foxa2 (Green) and nuclei (blue) on the uterine sections of *Cdo* KO and WT mice at PND 9, PND 12, PND 15 and PND 28. (b) Statistical analysis of the number of Foxa2-positive glands in the uterus of *Cdo* KO and WT mice at the stages examined. n ≥ 3. WT: wild type. KO: *Cdo* KO. PND: Postnatal day. Scale bar represents 50 μm.

In order to investigate the effect of *Cdo* KO on uterine gland formation and development, IF staining for Foxa2, a specific marker of GE, was performed since from PND 9 as epithelial invaginations become evident between PND 7 and PND 9. In WT mice, a few Foxa2-positive uterine glands were observed at PND 9, with a marked increase at PND 12, 15 and 28 (Fig 4a). However, in *Cdo* KO mice, no Foxa2-positive cells was detected at PND 9, and only a few uterine glands could be observed at PND 12 and PND 15 (Fig 4a). By PND 28, a small number of uterine glands were detected in *Cdo* KO mice, but this was significantly reduced compared to WT mice. In addition, quantitative analysis showed that the number of the uterine glands in *Cdo* KO mice at PND 28 was reduced by approximately 60% than in WT mice (Fig 4a and b). These findings demonstrate that *Cdo* KO impairs uterine gland development, and suggest that CDO or taurine are essential for the proper formation and development of uterine gland.

### 5. Effects of *Cdo* KO on cell proliferation and apoptosis in mouse uteri

**Fig 5:**
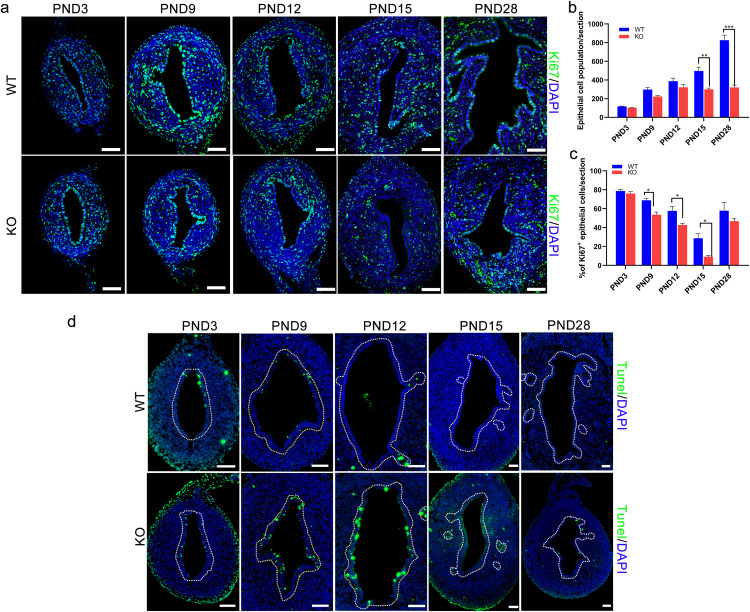
Effects of C*do* KO on cell proliferation and apoptosis in mouse uteri. (a) IF staining of Ki67 (Green) and nuclei (blue) on the uterine sections of *Cdo* KO and WT mice at PND 3, PND 9, PND 12, PND 15 and PND 28. (b) Statistical analysis of the number of Ki67 positive cells in the uterus LE of *Cdo* KO and WT mice. (c) Statistical analysis of the percentages of Ki67 positive cells accounting for the total epithelium cells of the *Cdo* KO and WT mice. n ≥ 3. (d) TUNEL detection (green) of the uterus tissue of *Cdo* KO and WT mice at PND3, PND 9, PND 12, PND 15 and PND 28. WT: wild type. KO: *Cdo* KO. PND: Postnatal day. Scale bar represents 50 μm. ***P < 0.001, **P < 0.01, ***P < 0.001.

As *Cdo* KO markedly decreased the taurine level in the uterus and impaired the formation and development of uterine gland, we further examined its effects on cell proliferation and cell apoptosis during uterine development. To evaluate cell proliferation, Ki67 staining was performed as a marker for proliferating cells. It was observed that Ki67-positive cells were broadly distributed throughout the uterine tissues (Fig 5a). Quantitative analysis showed no significant differences in the number or percentage of Ki67-positive cells relative to total epithelial cells between *Cdo* KO and WT uteri at PND 3 (Fig 5a–c). However, from PND 9 to PND15, the percentage of Ki67-positive epithelial cells was significantly lower in *Cdo* KO uteri compared to WT uteri (Fig 5a–c). By PND 28, no significant differences were observed between the two groups (Fig 5c).

Furthermore, in order to identify whether *Cdo* KO impaired uterine development by increasing uterine cell apoptosis, TUNEL analysis was carried out on uterine tissues at PND3, PND 9, PND 12 and PND 28. The results showed that TUNEL IF staining was not significant in the uterine epithelium of *Cdo* KO and WT mice at PND 3 (Fig 5d), but the TUNEL positive signals in the uterine luminal epithelium of *Cdo* KO mice at other stages examined were much stronger than in WT mice (Fig 5d). These findings suggest that the developmental defects and hampered glandular formation in *Cdo* KO uteri are closely associated with reduced epithelial cell proliferation and increased apoptosis in uterine epithelium.

### 6. Taurine supplementation partially rescues impaired uterine development and gland formation caused by *Cdo* KO

**Fig 6:**
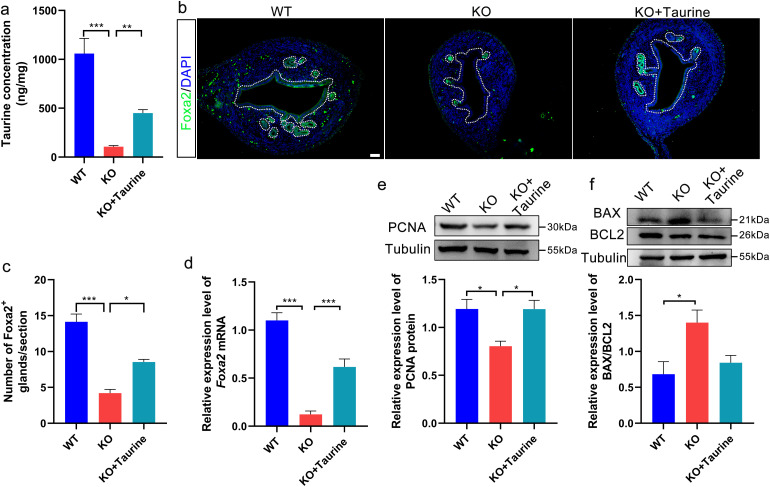
Taurine supplement partly recues the impairment of uterine development and gland formation caused by *Cdo* KO. (a-d) 10 days taurine supplementation to the *Cdo* KO mice (KO+Tau) increases the taurine level (a), the number of uterine glands marked by *Foxa2* (b, c) and Foxa2 mRNA levels (d) in the uterine tissue. (e) Western Blot detection and analysis of PCNA protein levels in the uteri of the WT, *Cdo* KO and KO + Tau mice (n ≥ 3). (f) Western blot detection of BAX/BCL2 protein levels (up) and the analysis of BAX/BCL2 ration (down) in the uteri of the WT, *Cdo* KO and KO + Tau mice. WT: wild type. KO: *Cdo* KO. PND: Postnatal day. Scale bar represents 20 μm. *P < 0.05, **P < 0.01, ***P < 0.001.

As the maintenance of the global taurine level relies on the endogenous taurine synthesis through the action of CDO and active dietary uptake, taurine supplementation was administered to the *Cdo* KO mice from PND 5 by intraperitoneal injection (500 mg/kg/day) for 10 days. Uterine taurine levels were then assayed at PND 15. The results showed that taurine levels in uterine tissues of taurine-supplemented *Cdo* KO mice (KO + Tau) were significantly higher than that in untreated *Cdo* KO mice, although they remained significantly lower than that of WT mice (Fig 6a). Further, in order to determine whether taurine supplementation could alleviate the effects of *Cdo* KO on cell proliferation and apoptosis during uterine development, Foxa2 staining was conducted. The results showed that the number of the uterine glands marked by Foxa2 in KO + Tau mice was significantly greater than that in *Cdo* KO mice, although it was still fewer than in WT mice (Fig 6b and c). This was further confirmed by a substantial increase in *Foxa2* mRNA levels in the uteri of *Cdo* KO mice upon taurine supplementation (KO* *+ Tau) (Fig 6d).

In addition, we assessed the expression of PCNA, BAX and BCL2 proteins in the uterus by Western blot. The results showed that taurine supplementation restored uterine PCNA levels in *Cdo* KO mice to the levels observed in WT mice (Fig 6e), and reduced the elevated BAX/BCL2 ratio in *Cdo* KO mice by 40% (Fig 6f). These results demonstrate that exogenous taurine supplementation partially rescued the defects of uterine development and adenogenesis induced by *Cdo* KO and taurine deficiency. They further suggest that taurine plays important roles in regulating uterine development and adenogenesis by modulating cell proliferation and apoptosis.

## Discussion

Extensive research has explored uterine development and adenogenesis, with most studies focusing on the intrinsic regulatory factors, such as developmental genes and transcription factors and their related mechanisms [31]. However, little is known about the role of nutrients, particularly taurine, in uterine development and adenogenesis. Using the *Cdo* KO mouse model, this study provides the first evidence demonstrating that *Cdo* KO results in taurine deficiency and the defects of uterine development and gland formation, although the broader systemic effects of *Cdo* KO on the general weight and other organ has been reported [28]. In addition, the abnormalities of uterine development in *Cdo* KO mice can be partially rescued by taurine supplementation. These findings demonstrates that CDO and taurine are crucial for prepubertal uterine development and adenogenesis in mice.

The uterine adenogenesis of mouse occurs from PND 3 to PND 21 [11,12], which is independent on steroids. Mouse uteri then turn into rapid growth through massive cell proliferation mainly regulating by steroids [2,12]. This is in accordance with our results here that the ratio of Ki67 positive cells and total epithelial cell population significantly increase in Ki67 positive cells. While uterine adenogenesis of mouse is a typical process of branching morphogenesis, requiring active cell proliferation of epithelium and the LE–GE differentiation, both being the key events in uterine morphogenesis and gland development [29,32]. Our results show that *Cdo* KO and/or taurine deficiency results in the reduction of cell proliferation, as indicated by a significant decrease in Ki67-positive epithelial cells during the uterine adenogenesis. In contrast, apoptosis, as detected by fluorescence TUNEL staining, is markedly increased in the uterine epithelium in *Cdo* KO mouse. Consistently, PCNA protein levels significantly decline, whereas BAX expression level and BAX/BCL2 ration are sharply elevated in the uterus of *Cdo* KO mouse. This pathological imbalance between cell proliferation and cell apoptosis caused by *Cdo* KO may disrupts the epithelial cell population growth and impairs uterine gland formation. While the increase in the BAX/BCL2 ratio suggests an apoptotic shift, it alone does not fully define the apoptotic cascade. However, given that BAX activation is a prerequisite for mitochondrial outer membrane permeabilization and subsequent caspase activation [33,34], these results provide evidence that apoptosis occurs via the intrinsic pathway. Future studies examining caspase-3 and PARP cleavage would help further confirm the mechanistic link between mitochondrial dysfunction and DNA fragmentation, further refining our understanding of CDO and taurine’s role in apoptosis regulation.

These findings presented here align with previous reports showing that the formation of epithelial branching systems is accompanied with massive cell proliferation, while the organ itself also expands significantly in size as it is being built [12,35]. In wild-type mice, both LE and GE cells express CDO, and FOXA2-positive GE cells and epithelial glands progressively increased in the study period, paralleling the rise in uterine CDO expression. Notably the increase in uterine CDO expression occurs concurrently with the elevation of Foxa2- positive GE cells and epithelial glands, and all of LE and GE cells express CDO. However, in *Cdo* KO mice, proliferation is significantly inhibited, and the number of FOXA2-positive GE cells is markedly reduced. Of note, taurine supplementation partially recues these defects caused by *Cdo* KO and taurine deficiency, suggesting that CDO and taurine are crucial for uterine adenogenesis by promoting the LE-GE differentiation.

In addition, it has been reported that CDO is highly expressed in the uterus of adult mice, and both uterine tissue and ULF are rich in taurine [30]. Our study further extends these findings by showing that uterine CDO expression and taurine concentrations progressively increase from PND3 to PND 28, suggesting that the mouse uterus acquires the ability to synthesize taurine postnatally. However, the impact of *Cdo* KO-induced defects in uterine development and adenogenesis on the reproductive capacity remains to be fully clarified.

Collectively, the present study provides the first characterization of the ontogeny of uterine CDO expression from PND 3 to prepuberty and its strong correlation with uterine taurine levels. Our findings show that *Cdo* KO and the resulting taurine deficiency impair the formation and development of uterine gland by inhibiting epithelial proliferation and increasing apoptosis, which could be partially rescued by exogenous taurine supplementation. Furthermore, we show that the mouse uterine tissue acquires the ability to synthesis taurine, highlighting CDO and taurine as crucial factors regulating the uterine adenogenesis. These results suggest that taurine could be a potential therapeutic agent for improving reproductive efficiency in livestock industry and also human reproductive medicine, although further research is needed to elucidate the precise mechanisms underlying the role of CDO and taurine in uterine development and their broader implications for reproductive health.

## Supporting information

S1 File Original Western blot and gel electrophoresis images. (DOCX)

S2 File Experimental data used for statistical analysis. (XLSX)
