# Supplementary figures and images for: Cysteine dioxygenase knockout and taurine deficiency impair mouse uterine adenogenesis by inhibiting epithelial cell proliferation and enhancing apoptosis
Source: PLoS One. 2025 Aug 18;20(8):e0329503. doi: 10.1371/journal.pone.0329503 (PMC12360574; doi:10.1371/journal.pone.0329503)

Original Blots images





Original Gel image (Fig.2a)


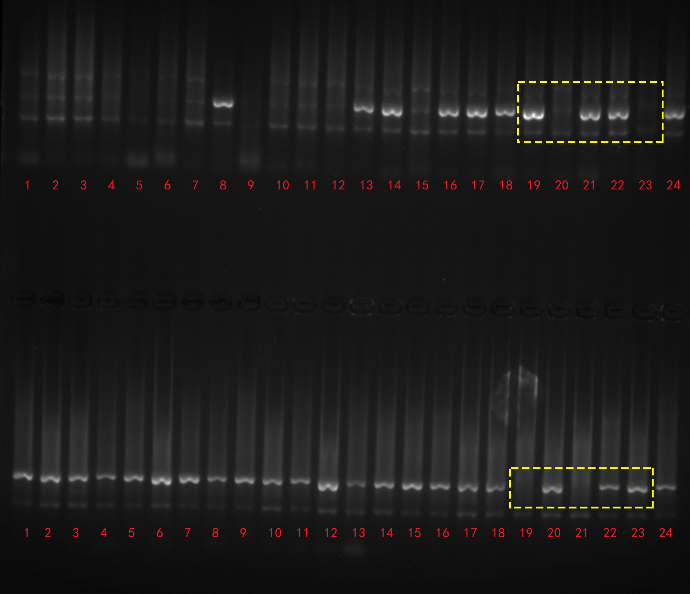

Supplement: S1 File — (DOCX) [file pone.0329503.s001.docx]
